# Pathological complete response following immunotherapy in dMMR/MSI-H ascending colon primary squamous cell carcinoma: a case report

**DOI:** 10.3389/fimmu.2026.1688501

**Published:** 2026-03-25

**Authors:** Chaoxian Xiong, Weimin Wang, Xuefeng Cha, Jie Li, Yifan He, Xiaoxia Li, Shulian Tang, Quan Yang, Kun Yu

**Affiliations:** Department of Colorectal Surgery, Yunnan Cancer Hospital, The Third Affiliated Hospital of Kunming Medical University, Kunming, China

**Keywords:** deficient mismatch repair, immunotherapy, microsatellite instability-high (MSI-H), pathological complete response (PCR), primary colon squamous cell carcinoma

## Abstract

Primary colon squamous cell carcinoma (SCC) is an extremely rare malignancy and associated with a poor prognosis. This case report describes a patient with deficient mismatch repair/microsatellite instability-high (dMMR/MSI-H) ascending colon SCC, who was treated at our institution. After receiving four cycles of programmed cell death -1 (PD-1) blockade monotherapy, the tumor exhibited significant regression, and pathological complete response (pCR) was achieved following surgical resection. This case demonstrates that Anti-PD-1 therapy can induce clinically meaningful tumor regression even in this rare colon SCC subtype, suggesting a potential treatment strategy for dMMR/MSI-H colon SCC. These findings may provide valuable insights for clinicians managing similar cases. However, the feasibility and safety of immunotherapy in dMMR/MSI-H primary colon SCC require further validation through additional clinical studies.

## Introduction

In recent years, the incidence of colorectal cancer (CRC) has been steadily increasing. In 2022, CRC accounted for over 1.9 million new cases and 904,000 deaths worldwide, representing nearly one-tenth of all cancer cases and fatalities, ranking as the third most common cancer and the second leading cause of cancer-related mortality globally (1). Adenocarcinoma is the most common histopathological type of CRC, while SCC of the intestinal tract typically originates in the anal canal and is extremely rare in the colon and rectum, comprising only 0.1-0.25‰ of all CRC, SCC occurs more frequently in the rectum and is even rarer in the colon (2). The first case of primary colon SCC was reported by Schmidtmann in 1919 (3). Compared to colon adenocarcinoma, primary colon SCC is more often diagnosed at an advanced stages and carries a worse prognosis (4). A study of 249 cases from the National Cancer Database(NCDB)revealed that 55.4% of primary colon SCC patients were female, suggesting a higher incidence in women. Furthermore, colon SCC is more frequently diagnosed at an advanced stages and has a poorer prognosis compared to rectal SCC (5).

**Figure 1:**
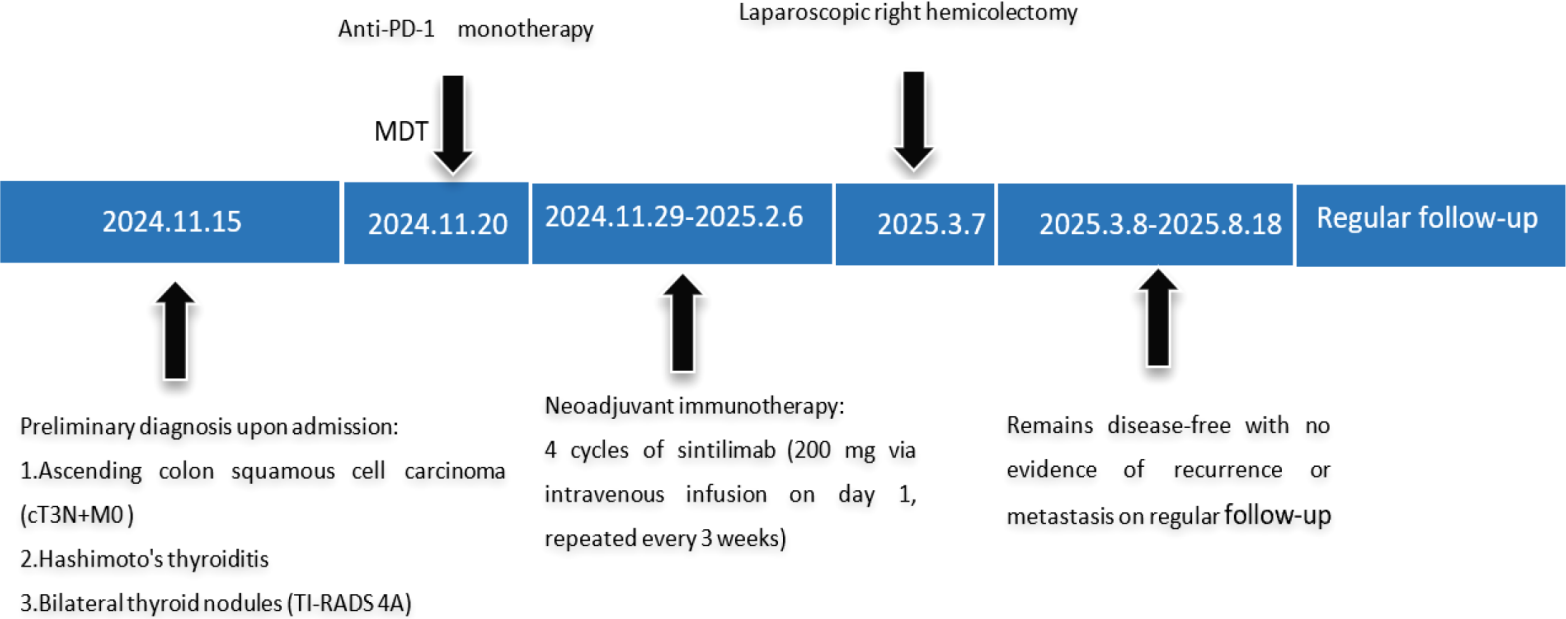
Treatment process flow chart for this case. MDT, multidisciplinary team.

Due to the rarity of colon SCC, no standardized treatment guidelines for colon SCC have been established. In currently reported cases, surgical resection of the primary tumor remains the mainstay of treatment (6), followed by individualized postoperative management. Immunotherapy for colon SCC has rarely been documented. Here, we systematically documented the complete therapeutic course of anti-PD-1 therapy for colon SCC. (**Figure 1**).

## Case introduction

**Figure 2:**
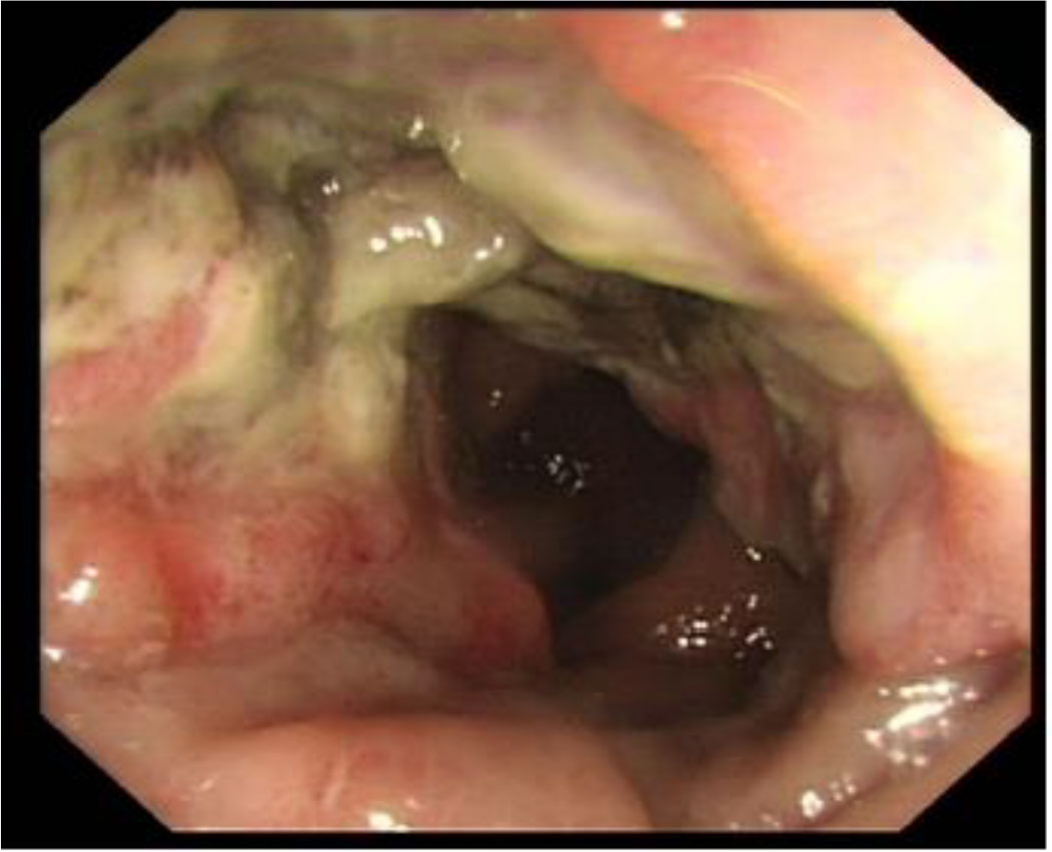
Preoperative colonoscopy identified a mass in the ascending colon.

**Figure 3:**
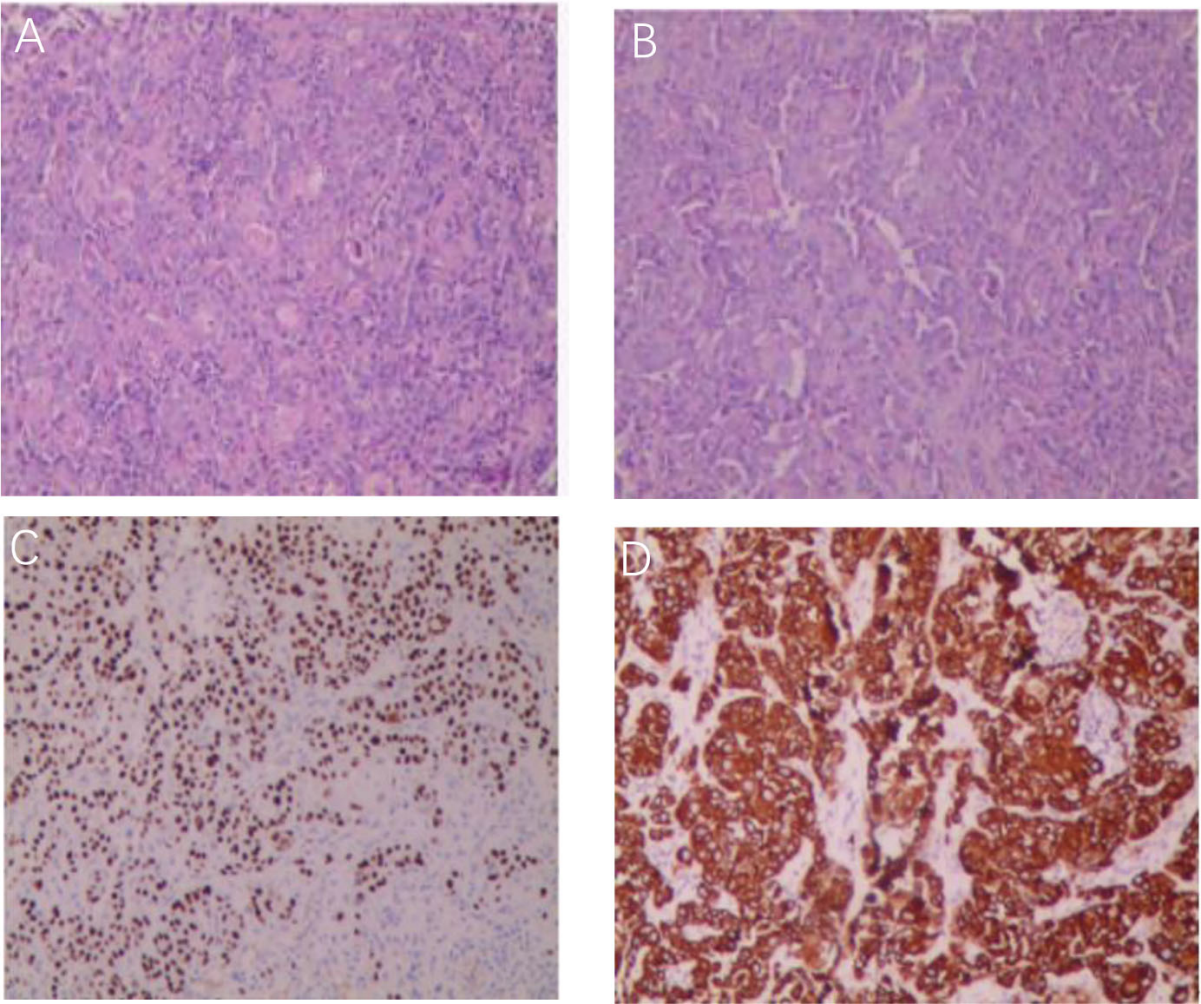
**(A, B)** The preoperative colonoscopic biopsy HE staining results of the tumor in the ascending colon confirmed SCC. Immunohistochemical results for P63/P40 **(C)** and CK5/6 **(D)**.

**Figure 4:**
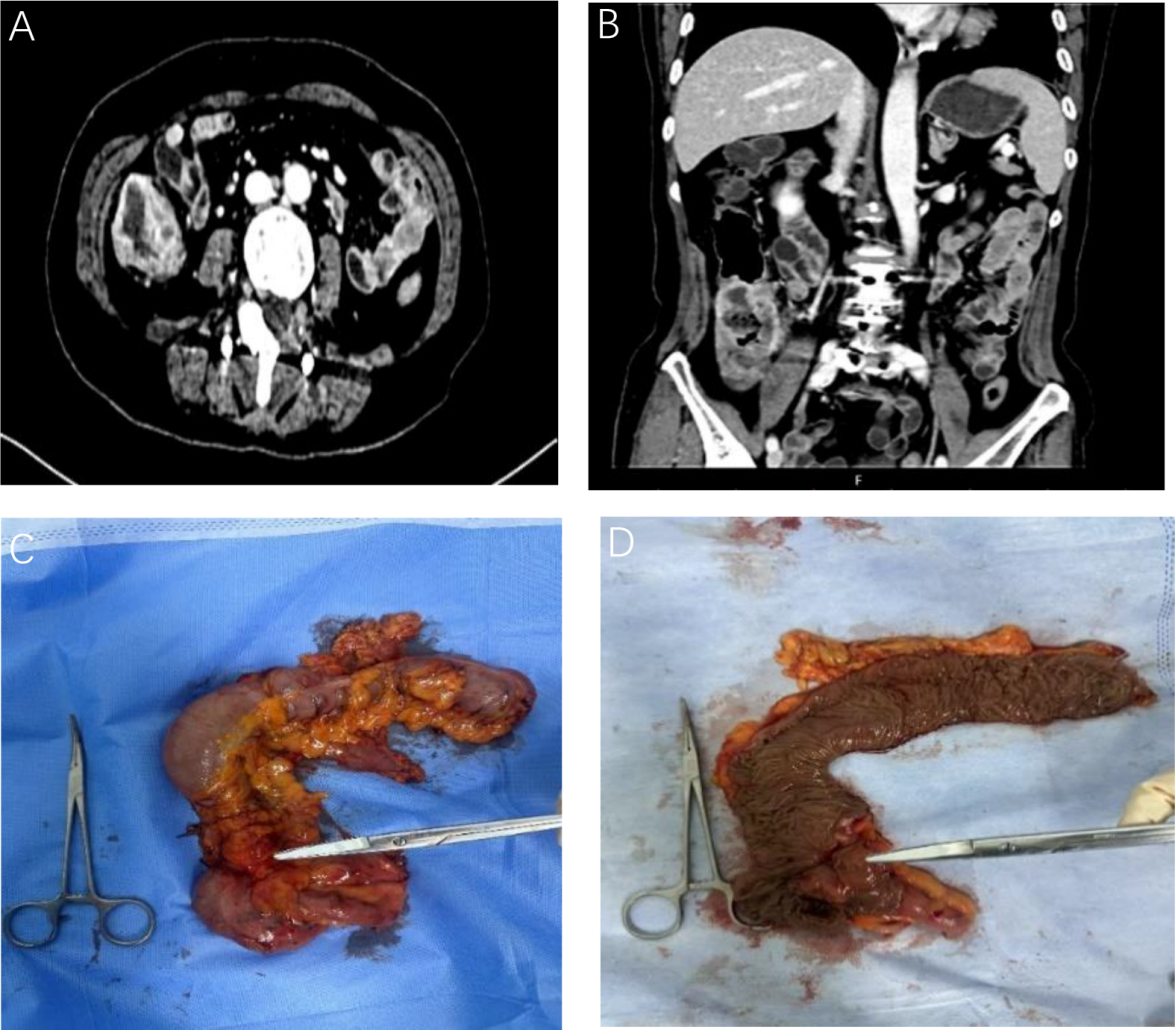
**(A, B)** Contrast-enhanced abdominal CT scans of the ascending colon tumor (November 13, 2024); **(C, D)** Gross pathological specimens of the resected tumor postoperatively.

A 74-year-old female patient was admitted on November 15, 2024, due to “abdominal pain accompanied by hematochezia for over one month.” Prior to admission, Initial evaluation at a local hospital revealed an ascending colon mass on colonoscopy, with biopsy confirming SCC. Her surgical history included lumbar spondylolisthesis repair and hysterectomy. Physical examination at our institution showed a soft abdomen with a 10cm midline scar, no tenderness or palpable masses, and unremarkable digital rectal examination (6cm insertion depth, smooth mucosa, no blood). Repeat colonoscopy at Yunnan Cancer Hospital identified an irregular circumferential ulcerated tumor involving the ileocecal valve (**Figure 2**).The biopsy pathology report confirmed the diagnosis of ascending colon SCC, Immunohistochemical results were:MLH1(+), MSH2(+), MSH6(+), PMS2(-). P63(+), P40(+), CK5/6(+), CK(+),CK7(-), CK20(-),VILLIN(-), CDX-2(partial+), KI67(50%+), S100(-), confirming moderately-to-poorly differentiated SCC (**Figure 3**). Immunohistochemical analysis revealed that the tumor cell nuclei showed positive expression for MLH1, MSH2, and MSH6, while PMS2 expression was negative. This pattern is consistent with isolated loss of PMS2 protein expression, which is a typical manifestation of dMMR. Contrast-enhanced CT (**Figures 4A, B**) revealed circumferential wall thickening involving the full thickness of the distal ascending colon, highly suggestive of malignant involvement, with multiple suspicious peri-colonic lymph nodes, Contrast-enhanced CT of the rest of the body was unremarkable. All measured tumor markers were within normal reference ranges. Thyroid evaluation showed bilateral nodules (TI-RADS 4A) and biochemical evidence of Hashimoto’s thyroiditis (elevated thyroglobulin >500 ng/mL and anti-TPO antibodies 179.54 IU/mL). The head and neck surgery team assessed the thyroid nodules as benign, recommending surveillance. Genetic testing revealed MSI-H status in the tumor tissue. Preliminary diagnosis upon admission:1. Ascending colon squamous cell carcinoma (cT3N+M0); 2. Hashimoto’s thyroiditis; 3. Bilateral thyroid nodules (TI-RADS 4A).

A multidisciplinary team (MDT) discussion was conducted at Yunnan Cancer Hospital, the consensus was: Given the patient’s imaging, pathological and immunohistochemical findings, a diagnosis of primary colon SCC (cT3N+M0) was confirmed. Due to the rarity of colon SCC, no standardized treatment guidelines exist. However, based on the dMMR/MSI-H status identified through immunohistochemistry and genetic testing, neoadjuvant therapy with a Anti-PD-1 monotherapy was recommended for tumor downstaging prior to surgery. After thorough discussion with the patient and family members regarding the potential benefits and risks of this approach, the decision was made to proceed with neoadjuvant Anti-PD-1 therapy. The patient received four cycles of sintilimab (200 mg via intravenous infusion every three weeks) from November 29, 2024 to February 6, 2025. Upon re-evaluation on March 4, 2025, significant tumor regression was observed. After preoperative preparation and anesthesia assessment, the patient underwent laparoscopic right hemicolectomy on March 7, 2025. A comprehensive pathological examination of the postoperative specimen revealed the following: Primary Site (Right Colon and Tumor): No viable tumor cells were identified, with only post-treatment reactive changes observed (chronic inflammation with hemosiderin deposition). According to the Ryan Tumor Regression Grade (TRG) system, this was classified as Grade 0, indicating a pCR. Margins: Microscopic examination showed no evidence of carcinoma at all intestinal (proximal, distal) and circumferential (radial) resection margins. Lymph Nodes: All 29 lymph nodes examined were negative for metastasis (0/29).

Appendix: Chronic appendicitis was noted. Additionally, gross examination of the tumor specimen revealed a distinct area of focal fibrosis at the site of the original tumor (**Figures 4C, D**). Postoperatively, the patient recovered with total parenteral nutrition support and was discharged after suture removal. The MDT confirmed a pCR. In line with the patient’s preference, the patient elected not to proceed with adjuvant chemotherapy. The patient continues on regular outpatient surveillance, which includes periodic thyroid function monitoring, and has experienced no immune-related adverse events to date.

## Discussion

Colorectal SCC is an extremely rare disease. Currently, the etiology and pathogenesis of primary colorectal SCC remain unclear. Studies have reported that its development may be associated with ulcerative colitis, Entamoeba histolytica, schistosomiasis, and human papillomavirus (HPV) infection (7–9, 24). However, some literature indicate no clear association between HPV infection and colorectal SCC (10). Additionally, long-term chronic inflammatory stimulation (11), differentiation of pluripotent stem cells into squamous cells (12), malignant transformation of persistent ectopic embryonic nests of ectodermal cells (13), and previous radiotherapy history (14, 15) may all contribute to primary colorectal SCC. The occurrence of primary colon SCC may also be related to squamous differentiation of adenomas or adenocarcinomas (16, 17). In reported literature, patients with primary colorectal SCC have also presented with other types of tumors, such as prostate cancer (18), ovarian cancer (19), endometrial cancer (20), and breast cancer (21). In this study, the patient’s immunohistochemical results showed P63 (+) and CK5/6 (+), which are highly predictive of a primary tumor of squamous epithelial origin (22), but no related pathogenic factors mentioned above were identified.

Primary colon SCC shares similar clinical presentations with colon adenocarcinoma, with common symptoms including abdominal pain, hematochezia, changes in bowel habits, and weight loss. Some patients may develop complications such as intestinal perforation, hypercalcemia, or persistent leukocytosis (23), with symptoms typically lasting weeks to months. Due to the unclear underlying mechanisms and low incidence of primary colon SCC, the diagnostic criteria established by Williams et al. (24) are generally recognized: (1) exclusion of metastatic SCC from other primary sites; (2) ruling out SCC secondary to squamous epithelialization of fistulous tracts; (3) ruling out proximal extension of anal canal SCC (particularly rectal primary SCC); (4) histopathological confirmation of pure SCC without glandular differentiation. In our case, the patient presented with abdominal pain and hematochezia as initial symptoms, consistent with primary colon SCC. Pathological examination confirmed that the colonic lesion consisted exclusively of squamous cell components without glandular differentiation. Although the lack of a pre-treatment PET-CT scan precludes ruling out occult disease with maximal sensitivity, the integrated evidence from whole-body contrast-enhanced CT, colonoscopy, immunohistochemical profiling, and postoperative surveillance forms a coherent diagnostic chain that strongly affirms the diagnosis of primary colon SCC, fulfilling all diagnostic criteria for primary colon SCC.

Due to the rarity of primary colorectal SCC, there are currently no standardized treatment guidelines, as randomized controlled trials are lacking. In reported cases, surgical resection of the primary tumor is typically performed for resectable disease, followed by individualized postoperative management. For advanced or metastatic cases, multimodal palliative approaches including chemoradiotherapy are employed (25).The application of immune checkpoint inhibitors (ICI) in colon SCC has been rarely reported. However, recent advances have demonstrated the significant potential of immunotherapy in colorectal cancer, particularly for MSI-H/dMMR patients, who represent 15-20% of all colorectal cancer cases (26), this molecular profile profoundly influences the tumor’s biological behavior and therapeutic response, leading to distinct treatment characteristics compared to other phenotypes of colorectal cancer (27).Substantial clinical evidence indicates that patients with dMMR/MSI-H colon carcinoma exhibit intrinsic resistance to traditional chemotherapy regimens based on 5-fluorouracil (5-FU) and oxaliplatin. These patients derive minimal, if any, benefit from 5-FU-containing adjuvant chemotherapy and may even be harmed by it (28). This resistance is even more pronounced in the neoadjuvant setting. Patients with locally advanced dMMR/MSI-H colorectal cancer gain limited benefit from neoadjuvant chemoradiotherapy (nCRT) or neoadjuvant chemotherapy (nCT), achieving a very low pCR rate of approximately 7%, which is significantly lower than that of pMMR patients (29). In stark contrast to these historical treatment failures stands the successful achievement of pCR in the primary colon SCC of the present case. The pCR achieved in this case, with its unique pathological features, provides microscopic evidence for the specific action of immunotherapy. According to the concept of immune-related pathological response criteria (irPRC), tumor regression following immunotherapy is characterized not only by the disappearance of tumor cells but also by the formation of a distinctive “immune regression bed” (30). The fibrosis, chronic inflammatory infiltration, and hemosiderin deposition observed in this case collectively constitute the typical hallmarks of such a regression bed. Fibrosis represents the endpoint of tissue repair, chronic inflammation suggests ongoing immune surveillance, and hemosiderin deposition is direct evidence of prior immune cell attack causing tumor vascular damage and hemorrhage, followed by phagocytic clearance (31). These features differ significantly from the pathological pattern of spontaneous tumor regression, which typically lacks such active inflammatory reaction and tissue remodeling traces. In summary, these irPRC-related pathological findings strongly support the notion that the pCR in this case was specifically mediated by a systemic anti-tumor immune response activated by immune checkpoint inhibitors. This not only directly corroborates the efficacy of the treatment regimen but also enhances the clinical significance of the case. In the context of neoadjuvant immunotherapy for dMMR/MSI-H colorectal cancer, early data strongly suggest that patients achieving pCR have an extremely low risk of recurrence. A systematic review indicated that disease-free survival rates exceeded 98% in most cohorts over a median follow-up of 8 to 26 months (32). Achieving pCR not only portends a favorable oncological prognosis but also influences subsequent treatment decisions, prompting clinicians to re-evaluate the necessity of postoperative adjuvant therapy. An international multicenter retrospective study on metastatic dMMR/MSI-H colorectal cancer showed that discontinuing immunotherapy after one year in patients who achieved a complete response (CR) did not negatively impact overall survival (33). In line with this evidence and considering the patient’s preference, no adjuvant chemotherapy was administered postoperatively in this case.

The successful use of ICI in dMMR/MSI-H CRC has fundamentally transformed the therapeutic paradigm for these patients. The KEYNOTE-016 study published in *The New England Journal of Medicine* in 2015 (34)first established the efficacy of pembrolizumab (a PD-1 inhibitor) in dMMR metastatic colorectal cancer (mCRC), marking the dawn of immunotherapy for CRC. Subsequently, the KEYNOTE-177 trial demonstrated that pembrolizumab significantly improved progression-free survival (PFS) compared to chemotherapy in first-line treatment of MSI-H/dMMR mCRC, with a median PFS of 16.5 months versus 8.2 months in the chemotherapy group. The objective response rate (ORR) and complete response (CR) rates were 43.8% and 11% in the pembrolizumab group, respectively, compared to 33.1% and 4% in the chemotherapy group, with fewer treatment-related adverse events observed. This pivotal trial led to the approval of pembrolizumab in China for first-line treatment of dMMR/MSI-H mCRC, earning a Category 1 recommendation in the CSCO CRC guidelines (35, 36).

The success of immunotherapy in mCRC has prompted its exploration in earlier disease stages, with promising results (37, 38). Both metastatic and non-metastatic dMMR/MSI-H CRC patients may derive clinically meaningful tumor regression and improved long-term outcomes from immunotherapy, potentially making it a preferred option. However, none of these studies included patients with primary colon SCC. Furthermore, some studies suggest that rectal SCC should be managed similarly to anal SCC (39), In patients with locally recurrent or metastatic anal SCC, a recent global phase III randomized controlled trial showed that adding retifanlimab (a PD-1 inhibitor) to standard chemotherapy (carboplatin and paclitaxel) improved overall survival(OS) and PFS with manageable toxicity (40), further underscoring the potential role of immunotherapy in SCC. To date, only a few case reports have described the use of immunotherapy in colon SCC, all involving proficient mismatch repair/microsatellite stable (pMMR/MSS) tumors as exploratory adjuvant therapy (41–44), with favorable outcomes reported. These findings highlight the need to investigate novel combination strategies to overcome resistance to anti-PD-1/PD-L1 therapy in MSS SCC and enhance treatment efficacy. However, there have been no reported cases of neoadjuvant immunotherapy monotherapy for MSI-H/dMMR colorectal SCC, primarily due to the extreme rarity of this patient population. This raises an important clinical question: Is immunotherapy equally effective in this rare subset of patients? In our case, the patient achieved a pCR following neoadjuvant Anti-PD-1 monotherapy and subsequent surgical resection, and at the time of writing, the patient showed no signs of recurrence or metastasis, and no immune-related adverse events were observed, confirming its safety.

This study has the following limitations. First, HPV testing was not performed, precluding exploration of a potential association between HPV infection and the development of colorectal SCC. Furthermore, given that this is a retrospective case report and treatment decisions were primarily based on dMMR/MSI-H status, data on PD-L1 expression and tumor mutational burden (TMB) were not available. This limits our ability to further investigate the relationship between these biomarkers and the pCR.

In summary, primary colon SCC is an exceptionally rare malignancy with unclear etiology and pathogenesis, requiring pathological and immunohistochemical confirmation for diagnosis. Currently, no standardized treatment guidelines exist. Our case demonstrates that immunotherapy may also benefit patients with MSI-H/dMMR primary colon SCC, inducing clinically significant tumor regression. This finding provides a valuable reference for managing similar cases in the future.

## Data Availability

The original contributions presented in the study are included in the article/supplementary material. Further inquiries can be directed to the corresponding authors.
